# Effect of a Home-based Exercise Program on Shoulder Pain and Range of Motion in Elite Wheelchair Basketball Players: A Non-Randomized Controlled Trial

**DOI:** 10.3390/sports7080180

**Published:** 2019-07-24

**Authors:** Saleky García-Gómez, Javier Pérez-Tejero, Marco Hoozemans, Rubén Barakat

**Affiliations:** 1 Sanitas Foundation Chair for Inclusive Sport Studies, Faculty of Physical Activity and Sports Sciences, Universidad Politécnica de Madrid, 28040 Madrid, Spain; 2 Faculty of Behavioural and Movement Sciences, Neuromechanics, Vrije University, 1081 Amsterdam, The Netherlands; 3 AFIPE Research Group, Faculty of Physical Activity and Sports Sciences, Universidad Politécnica de Madrid, 28040 Madrid, Spain

**Keywords:** adapted sport, Paralympic sport, wheelchair injuries, shoulder biomechanics, prevention, exercise

## Abstract

The aim of the present study was to assess the effects of a 10 week shoulder home based exercise program (SHEP) on shoulder pain (SP) and range of motion (ROM) in a group of elite wheelchair basketball (WB) players. A convenience sample of elite WB players (n = 36, 15 males and 21 females), mean age of 26 years (SD 7.6, range 15–45)) were assigned to either an exercise or a control group, according to the use of the wheelchair during daily activities. The shoulder pain index for wheelchair basketball players (SPI-WB), functional tests and ROM were measured at baseline and after a 10 week intervention. In the analysis of the SPI-WB scores, for the exercise and control groups separately, there were no significant reductions of SPI-WB scores after intervention. Related to the analysis between groups after 10 weeks of intervention, there were no significant differences in changes between the exercise and control groups (Z = 0.840, *p* > 0.05, r = 0.743). In this regard, there was a significant change after the intervention for shoulder extension ROM (Z = 2.81, *p* ≤ 0.05, r = 0.249). Shoulder Pain did not increase along the 10 weeks of the SHEP development in WB players who reported SP before the intervention program. However, in those players who started the intervention without SP, as no increase in SP was observed and players were free of injury. An exercise program could be a tool to maintain shoulder health and prevent injuries in elite WB players.

## 1. Introduction

Shoulder disorders are a common problem in wheelchair users [1,2]; a huge proportion of them experience shoulder pain (SP) [2,3,4,5,6,7]. SP is one of the most common symptoms of physical dysfunction caused by increased shoulder load and the repetitive stress of wheelchair handling [7,8,9]. In elite wheelchair athletes, upper limb injuries are frequent, with shoulder injuries being common [10,11]. The risk for SP further increases when wheelchair users also participate in wheelchair basketball (WB), which is the most popular adapted sport and is practiced worldwide [12]. In previous studies, about 72–85% of WB players reported SP related to activities of daily living (ADL) or sports activities [5,13]. In addition, playing WB involves movements such as pushing, breaking or turning the wheelchair [14,15]. Moreover, overhead movements involved while shooting, rebounding or executing long passes may promote SP [3,16,17].

According to prior research, elite WB players report many injuries such as ischial deep tissue injury [18,19] and upper limb muscle strength alterations [20]. Also, a high prevalence of rotator cuff pathology was found [21], and a difference in the risk of SP by gender [14,22]. A systematic review identified limited evidence in relation to shoulder injuries when performing overhead sports [23]. Also limited information related to the effect of shoulder injury prevention measures was found [24]. In this line, prevention strategies need to be implemented and coordinated with upper extremity evaluation and screening [25].

Furthermore, it has been shown that encouraging shoulder stability and mobility are fundamental training contents to prevent postural changes related to muscular imbalance. For these reasons, an intervention program would be useful for SP prevention in WB players. In this regard, many studies have been developed to analyze wheelchair users [26,27,28,29,30]. However, it is relevant to address that no research, to our knowledge, has been carried out that deals with the impact of an exercise program for elite WB players.

Previous studies [28,29] report the effectiveness of exercise protocols to reduce SP in wheelchair users, these researches included wheelchair athletes, however, they were not specific to WB players. Only one study focused on the evaluation of shoulder injury in collegiate WB players, explaining the influence of an exercise program on shoulder internal/external rotation [31]. Both the biomechanical assessment and the training program proved to be well perceived by junior WB athletes in training conditions [32]. In this regard, a shoulder maintenance program focused on adductors, external rotator and scapular retractors is important to keep shoulder muscles well-balanced in wheelchair athletes [27,33]. However, we did not find any research dealing with the application of a shoulder exercise program in WB players in preparation for an international competition. This information is important for physiotherapists and coaches in order to develop specific training programs for players’ shoulder health assessment and screening. Also, it is relevant to consider the role of sport participation on the shoulders of wheelchair users, as it could assist in the development of a preventive exercise program [34].

The aim of the present study was to assess whether a 10 week shoulder home-based exercise program (SHEP), focused on SP and range of motion (ROM), could improve and maintain shoulder function in a group of elite WB players in their preparation for major competitions.

## 2. Method

### 2.1. Study Design

**Figure 1:**
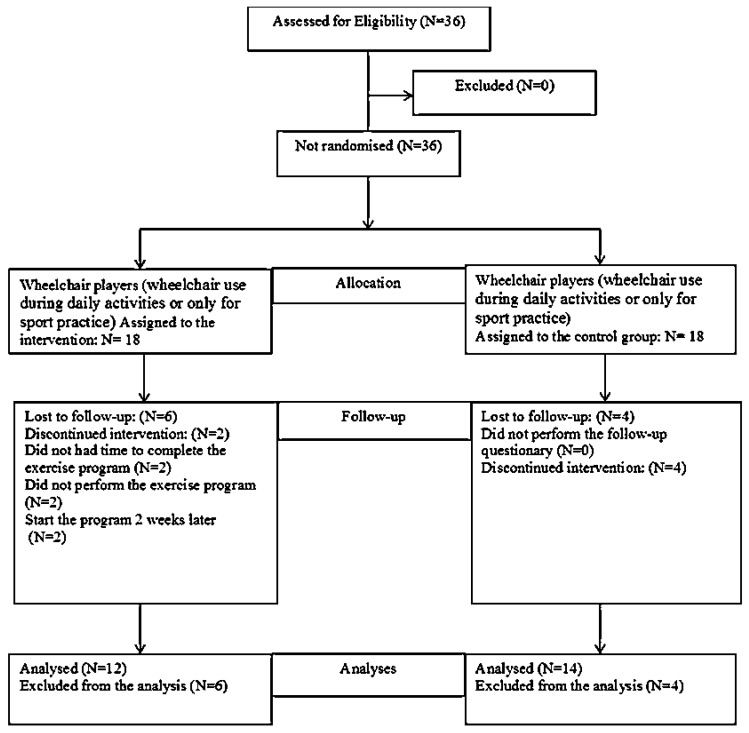
Flow chart showing how the participants moved through the study period.

The present study was a non-randomized clinical trial [35]. After baseline evaluation, participants were assigned, using a mixed-paired design, to either the exercise group (EG) or the control group (CG), taking into consideration two previous criteria, wheelchair use and SP. As such, each player was assigned to one paired group according to wheelchair use during daily activities and whether or not they had SP. For example, one player who uses a wheelchair for daily activities and one player who uses a wheelchair only for sports activity, with SP or without SP, were in each group. The procedure was performed by a researcher not engaged in the project, who assigned each participant according to previous criteria. The participants were either allocated to an intervention group, performing a resistance and stretching shoulder exercise program (n = 12), or a comparison group receiving the standard recommendation (n = 14) (Figure 1).

Assessments were performed at baseline and after 10 weeks. An ad hoc questionnaire was used to assess what kind of activities players carried out during the study. The physiotherapist responsible for the testing was blinded to the participants’ assignment, and players were told not to disclose their group. Data collection was performed throughout the training process in coordination with the teams’ technical staff. Participants were instructed to perform all exercises without taking any notice of their shoulder symptoms.

The study was conducted in accordance with the Declaration of Helsinki [36], and was approved by the Ethical Committee of the university. Also, the study was registered in Clinical Trial.gov (NC T02842008).

### 2.2. Participants

Thirty-six WB players, with a mean age of 26 years (SD 7.6, range 15–45, 15 males and 21 females), were recruited as a convenience sample from the pre-selection squad team of the national WB teams. The inclusion criteria for the participants were, that they were part of either the male or female pre-selection of the WB national teams, and that they possessed an official sport license at the time of the study. Also, participants were required to have used a wheelchair for at least one year before the study and to have at least one year of experience in competition. Moreover, for players using a wheelchair for daily activities, further inclusion criteria were, to use the wheelchair for at least three hours per day and perform at least six hours of WB practice per week. For players using the wheelchair only for WB practice, the additional criterion was to use the wheelchair for at least 6 h of wheelchair basketball training per week. Exclusion criteria for study participation were, presenting a history of acute injuries, shoulder dislocation and differential diagnoses in the year before the study. All participants signed informed consent. Parents were informed in cases where a participant was less than 18 years old. The study was developed over the four months before major competitions for both the female team (2016 Atri European Championship B, Italy) and the male team (2016 Rio de Janeiro, Paralympic Games, Brazil), in close collaboration with both national teams’ technical staff. WB players were identified and invited to participate in the study (Figure 1).

### 2.3. Intervention

The WB players allocated to the intervention group followed a detailed protocol including strengthening and stretching exercises in the upper extremities [26,27,28,29,30], along with general recommendations. In order to assure the quality and consistency of the SHEP, a qualitative analysis was developed based on the expert opinions of health and sport professionals, including a rehabilitation physician, a sport physician, physiotherapists and coaches [37].

Based on the information and evidence available in the previous literature, a SHEP (S1 in Supplementary Materials) was developed which focused on the prevention of shoulder injuries in WB players. The structure and content validity of this program were demonstrated [37] in previous studies [26,27,28]. The proposed program (S1 in Supplementary Materials) included:

- Warm-up exercises performed bilaterally in sagittal, coronal and transverse planes (eight repetitions of each).
- Five strengthening exercises performed for the serratus anterior, scapula retraction and depression, and shoulder rotation and adduction (three sets of ten repetitions, with 45 s of rest between sets). There was 10–15 s of rest between the stretches.
- Five stretching exercises performed for the trapezius superior, medial and inferior portion, posterior shoulder, pectoral and biceps brachial muscles (five repetitions of each, holding each stretch for 20 s, with rest for 15 s).

The warm-up, strengthening and stretching exercises were developed based on previous literature [26,27,28,29] and clinical experience. In this respect, an exercise program should include strength exercises for shoulder griddle stabilization muscles and strength optimization of the muscles used for pulling and pushing the wheelchair’s hand-rim.

The 10 week intervention program involved performing exercises three times per week at home, amounting to 36 sessions in total. The daily activities performed and adherence to the exercise program were assessed for each participant in each group every fourth week during the intervention period. To familiarize the players with the exercise program, a physiotherapist explained the exercise program after the first evaluation. 

The intervention program instructed participants to perform each session of exercise for 30 min. For a complementary description, see S1 in the Supplementary Materials. The exercise program was performed by each participant at home. All exercises were performed in a seated position. 

A hands-on instruction program was provided. Both the EG and the CG received a general protocol of postural recommendations (S2 in Supplementary Materials) as a standard care instrument. The research team provided support to all the participants in the EG and the CG via email throughout the study.

### 2.4. Outcome Measures

Outcomes were assessed before and after the 10 week intervention period in both the EG and the CG. The shoulder pain index for the wheelchair basketball players (SPI-WB) questionnaire was used to assess SP [38]. This index was based on the Wheelchair Shoulder Pain Index developed by Curtis et al. [3,4] and was found to be valid and reliable with a Cronbach score ∞ = 0.899, and a significant intraclass correlation coefficient (ICC) of r = 0.976. The questionnaire contained demographic data including an athlete’s lifestyle, fifteen items related to shoulder dysfunction and six items related to the respondent’s years of experience in WB, time since SP onset, SP location, numbness or cramps. Items related to pain during ADL (15 items) were also included, to distinguish between players who use the wheelchair for ADL (five specific items) and players who use the wheelchair only for sports activities (10 items). Also, four items related to SP perception when performing specific WB skills, including shooting, pushing the hand-rims, rebounding or one-handed long passes, and other game situations were included [13].

In addition, before and after the intervention, clinical tests were performed by a specific physiotherapist to diagnose orthopedic shoulder injuries. Neer’s sign and Hawkins–Kennedy tests were used to determine subacromial impingement [39,40]; these clinical diagnostic tests are commonly performed in clinical practice [41,42]. The Jobe test was also used to evaluate the integrity of the supraspinatus muscle and tendon [43,44], this being a simple technique that can improve the clinical diagnosis of rotator cuff tears [45]. In this line, the clinical test could provide support for a comprehensive clinical examination, being a starting point for clinical diagnosis of shoulder injuries. Finally, a single experienced clinician measured shoulder mobility (flexion, extension, abduction, external and internal rotation) using a medium plastic goniometer [46,47]. A single experienced clinician stabilized the shoulder ROM. For standardized goniometric measurements, intra- and inter-rater reliability was used prior to the study. A previous study determined the intra-rater (ICC from 0.60 to 0.95) and inter-rater reliability (ICC = 0.74) of the ROM assessment in a similar population [46].

### 2.5. Statistical Analysis

For descriptive statistics, data were checked for normality by exploring z-values for skewness and kurtosis, histograms, q-q plots, box plots and the results of the Shapiro–Wilks test for the EG and CG separately. For continuous variables, mean and standard deviation (SD) were determined, while for categorical variables, frequency and percentage (%) were used. T-tests, or their non-parametric equivalent for continuous data, or chi-square tests for categorical data, were used to compare the EG and the CG at baseline. The normality of the outcome variables (from baseline to 10 weeks) for the two groups was also checked by visual inspection of their histograms, q-q plots and box plots. Z-values of skewness and kurtosis as well as a Shapiro–Wilks test were carried out on the change scores as well. The data were assessed as not normally distributed, so Wilcoxon rank-sum tests were used to determine if there were differences between pre–post scores for each group. Also, a Mann–Whitney U test was used to determine if there were significant differences in change scores of the outcome variables between the two groups. In addition, a Spearman’s rank correlation test was calculated as a measure of effect size [48,49]. An r-value higher than 0.1, 0.3 or 0.5 was considered as a small, medium or large effect size, respectively [50]. Statistical analyses were performed with IBM SPSS Statistics 24.0 (IBM Corporation Armonk, New York, NY, USA) and *p* ≤ 0.05 was accepted as being significant.

## 3. Results

Eligible participants were recruited in the context of preparation for international competitions. Demographic characteristics of all the exercise and control groups’ participants at baseline are presented in Table 1. There were significant differences for the type of disability, level of injury, dominant side, SP and time of SP in the demographic characteristics at baseline.

Of the 18 WB players in the EG, only 12 were included in the SHEP intervention (seven males and five females). Two participants withdrew for personal reasons and did not complete the 10 weeks of the exercise program. Also, two participants started the program two weeks later. The remaining two participants were lost before the follow-up because they did not participate in the last evaluation session. At the fourth and eighth week, 58.3% of this sample showed adherence to the SHEP. In the CG, 14 participants were included (six males and eight females), four participants were lost before the follow-up from the clinical trial because they did not come to the final evaluation.

Regarding SP scores, in the EG, before the intervention, five (27.8%) players already presented SP and stayed unchanged after the intervention. In the CG, seven (41.2%) players showed SP at baseline; however, 11 (78.6%) players reported SP after 10 weeks.

In the analysis of the SPI-WB scores from pre- and post-tests, in both the EG and the CG, the SP score did not significantly change from baseline to post-intervention (*p* = 0.401 and *p* = 0.583, respectively). In relation to SP analysis between groups, there were no significant differences in changes between the EG and the CG after 10 weeks of intervention (Z = 0.840, *p* > 0.05, r = 0.743) (Table 2).

According to the clinical test, of the 12 participants in the EG, seven (58.3%) reported SP according to an impingement test detecting specific shoulder injuries before the intervention; however, two of them (16.7%) reported SP after the intervention. With respect to the CG, seven (50.0%) reported SP according to the clinical test detecting specific shoulder injuries before the intervention; however, six (42.9%) reported SP after the intervention.

For shoulder extension ROM, there were significant differences from baseline to 10 weeks in the EG (Z = 2.81, *p* ≤ 0.05, r = 0.249), decreasing 10° (SD 12) in extension, while in the CG a decrease of 5° (SD 11) was observed. There were no significant changes (*p* ≥ 0.05) after the intervention period in either group for flexion, abduction, internal and external rotation, with effect sizes varying from r = 0.596 to r = 0.960.

## 4. Discussion

The purpose of this study was to assess the effects of a 10 week SHEP on SP and ROM, to improve and maintain shoulder condition in world-class level WB players in the context of preparation for major competitions, such as the 2016 Atri (Italy) European WB Championship B for female teams and the 2016 Rio de Janeiro (Brazil) Paralympic Games for male teams. After SHEP intervention, WB players in the EG reported a reduction of SP according to SPI-WB scores; however, this change was not statistically significant. This finding is contrary to previous studies [26,27,28] that documented a significant change in wheelchair users after exercise intervention. However, these studies were not focused on maintaining the shoulder condition of elite WB athletes in an exigent training process. In relation to ROM, in general, participant players in the EG preserved their shoulder ROM after intervention, except for shoulder extension, where there was a reduction in ROM (Z = 2.81, *p* ≤ 0.05, r = 0.249). For the CG, there were no significant differences observed after the intervention, nor between groups.

In this study, SP did not significantly change after the intervention. A reason that might explain this finding could be the proportion of participants that reported SP before the SHEP intervention, of the total sample, seven players already presented SP. The difference between athletic and nonathletic populations may influence the results in different studies investigating SP. This could probably be because wheelchair athletes developed shoulder overload when performing sport movements during training sessions and competition, as well as during SHEP sessions [16,17]. Related to groups, there were no statistical differences between the EG and the CG for SP, in line with previous studies [26]. However, some studies showed significant differences in changes between groups related to SP in wheelchair users [27,29].

According to the results of clinical tests in our study, in the EG, seven players reported SP before the intervention; however, only two of them reported SP after the intervention. This could suggest an influence of the program in the reduction of the SP. On the other hand, in the CG, seven of the players showed positive clinical tests before the intervention; however, six reported SP after the intervention. In this regard, in the control group, there was a change in the incidence of SP.

Regarding ROM, no changes were found between groups (except for shoulder extension) after the intervention. In accordance with this, one study that determined the influence of an exercise program on shoulder ROM, specifically internal/external rotation in a specific sample of WB players, reported an association between more shoulder ROM and less SP in a pre–post test [31]. However, in our study, the participants maintained shoulder ROM, while extension ROM decreased after the exercise program. The tendency to maintain shoulder internal rotation and abduction ROM values was detected; this movement had a strong relationship with WB skills such as shooting and wheelchair propulsion.

It is relevant to emphasize that the SHEP focuses on shoulder condition maintenance, not on SP treatment for the characteristics of the population and the context of the data collection. This could be an important issue when comparing our results with previous studies [27,28,30]. In contrast with previous research [23,31,51], this study shows that a SHEP seems appropriate to prevent the development of SP and re-injury. In accordance with this, a study by García-Gómez et al. [37] reported evidence for the adequate structure and content validity of a SHEP according to expert opinions. The exercise program included special attention to front chest musculature and the back of the shoulder joint, which is often tight when the wheelchair user has a limited inward rotation of the shoulder [52].

Recently, literature reported that shoulder exercise programs may decrease SP and improve functionality in wheelchair users [52,53,54,55]. Furthermore, it has been shown that the results of these programs report that encouraging shoulder stability and mobility is fundamental to prevent postural changes and muscular imbalance. In this regard, periodization training for elite athletes with disabilities should include strength and flexibility exercises [56]. It is necessary to include an appropriate type of exercise with parameters of intensity (from 40 to 90 percent), frequency (two to three sets of 8–12 repetitions) and number of sessions per week (between two and four days per week, over a period of four to six months) [57,58]. According to previous authors, an exercise program should include a warm-up phase, resistance training, and stretching positions. Holding stretches for 15–30 s was suggested for flexibility training at shoulder level [26,27,28]. The SHEP periodization is useful to maintain ROM along the training period, which seems highly necessary when dealing with WB players that train for at least three hours a day. In this sense, there were no differences in total amounts of training time between players.

Some of the limitations related to this SHEP application were that a non-randomized allocation was performed. The analysis and interpretation of these findings should therefore be done with caution due to the small sample size. Participants included only a specific group of elite WB players who were in preparation for major competitions. On the other hand, this study may have been biased because the same tester who performed the follow-up also collected the data. However, at the time of post intervention evaluation, the tester did not have access to the data, similar to the methodology developed by Van Straaten et al. [27] and Mulroy et al. [28]. After the intervention, WB players were free of injury, even after such an exigent preparation process, this being a key fact to enable them to perform at top-class international competitions.

Taking into consideration the fact that wheelchair basketball is an adapted sport, it implies a high level of effort [59] and the players practice for many hours to improve their skills. Importantly, an emphasis on proper wheelchair positioning and biomechanics is likely to reduce the risk of musculoskeletal injuries [60]. 

The data of this study reveal several practical applications. First, WB players seem to require regular shoulder evaluations and exercise recommendation strategies to stabilize the shoulder joint and promote health in an adequate way to perform ADLs and sports activities. This study suggests that WB players require a follow-up in order to supervise their shoulder condition. Secondly, a SHEP intervention to maintain shoulder function should focus on flexibility and balance of the shoulder muscles. Finally, the multidisciplinary team should emphasize their actions that maintain shoulder condition, and the team needs to be coordinated to implement this kind of approach, which could be applied to both athletes and non-athletes with physical disabilities. Our study provides the scientific community with a maintenance strategy for this specific population of elite WB players to preserve shoulder function in a training period when preparing for a major competition.

## 5. Conclusions

The tested SHEP appears to be useful for maintaining shoulder conditions of WB players throughout their sport training regimes when preparing for elite WB competitions, such as the Paralympic Games. In a group of WB players who received a 10 week SHEP, the changes in SP and ROM were not significantly different from the changes observed in the CG that did not receive the intervention. For both groups, no increase in SP nor shoulder injuries was observed, so the functionality and the health of the shoulder were preserved. Further research is needed to determine the effectiveness of the SHEP in a larger sample, and also to orientate future steps to develop technological innovations and include prevention programs in the players’ preparation.

## Figures and Tables

**Table 1:** Baseline demographic characteristics of the participants (n = 36).

Characteristics		Exercise Group (EG) (n = 18)	Control Group (CG) (n = 18)	*p*
Gender	Male	(9)50%	(7)38.9%	0.505
	Female	(9)50%	(11)61.1%	
	Age (years)	27.11 ± 6.720	25.9 ± 7.615	0.650
Occupation	Administrative	(4)22.2%	(6)33.3%	
	WB Player	(4)22.2%	(5)27.8%	0.670
	Student	(6)33.3%	(5)27.8%	
	Others	(4)22.2%	(2)11.1%	
Functional Class	1–1.5	(4)22.2%	(3)16.7%	0.670
	2–2.5	(4)22.2%	(5)27.8%	
	3–3.5	(7)38.9%	(5)27.8%	
	4–4.5	(3)16.7%	(5)27.8%	
Type of Disability	Post-polio	(2)11.1%	0	
	Amputation	0	(2)11.1%	
	Others	(4)22.2%	(6)33.3%	
Level of Injury	T2-7 (high paraplegia)	(8)72.7%	(6)60%	*p* < 0.001
	T8 and below (low paraplegia)	(3)27.3%	(4)40%	
Wheelchair Users	Daily	(11)61.1%	(12)66.7%	0.096
	Only for Sport	(7)38.9%	(6)33.3%	
Dominant Side	Right	(16)88.9%	(15)83.3%	*p* < 0.001
	Left	(2)11.1%	(3)16.7%	
	Years since disability	18.28 ± 90398	16.95 ± 8.794	0.628
	Years of federated sport	11.83 ± 9.532	10.28 ± 8.428	0.743
	Years of recreative sport	10.28 ± 8.428	8.86 ± 8.013	0.542
Shoulder Pain	Yes	(5)27.8%	(7)41.2%	0.046
	No	(13)72.2%	(10)58.8%	
Time Duration of Shoulder Pain	One week	(1)20%	(1)14.3%	0.050
	Two weeks	(1)20%	(1)14.3%	
	More than 12 weeks	(3)60%	(5)71.4%	

Abbreviations: EG: exercise group; CG: control group. Values are mean (SD) for continuous variables, frequency (%) for categorical variables. The chi-square test was used for categorical variables, and the Mann–Whitney U test was used for continuous variables.

**Table 2:** Shoulder pain (SP) and range of motion (ROM) scores at baseline and post-intervention assessments.

Outcome Measures		EG (n = 12)	*p*-Value of Change Over Time	CG (n = 14)	*p*-Value of Change Over Time	*p*-Value of Differences between Groups in Changes Over Time	Effect Size
Shoulder Pain Index for Wheelchair Basketball players (SPI-WB)	Baseline	6.30(32)		6.60(23)			
	Post-Intervention	3.75(12)	0.401	10(19)	0.583		
	Change	0(24)		0(22)		0.742	0.743
Flexion ROM	Baseline	180(8)		180(0)			
	Post-Intervention	173.75(17)	0.056	177.50(13)	0.575		
	Change	2.50(18)		0(7)		0.860	0.859
Extension ROM	Baseline	71.25(9)		62.50(14)			
	Post-Intervention	60(12)	0.005	60(0)	0.288		
	Change	10(12)		5(11)		0.252	0.249
Abduction ROM	Baseline	180(35)		180(0)			
	Post-Intervention	177.50(16)	0.672	180(9)	0.498		
	Change	0(24)		0(9)		0.940	0.916
Internal Rotation ROM	Baseline	71.25(20)		66.25(13)			
	Post-Intervention	71.25(23)	0.368	70(13)	0.212		
	Change	−1(12)		0(13)		0.980	0.960
External Rotation ROM	Baseline	90(0)		90(8)			
	Post-Intervention	90(0)	0.593	90(3)	0.458		
	Change	0(0)		0(2)		0.667	0.596

Abbreviations: EG: exercise group; CG: control group. Values are median (range) for continuous variables. *p*-values for the change scores of the outcome variables between the two groups were obtained using Mann–Whitney U test. Within-group *p* was obtained using the Wilcoxon test.

## Supplementary Materials

The following are available online at https://www.mdpi.com/2075-4663/7/8/180/s1, S1: Shoulder exercise program to prevent shoulder injuries in wheelchair basketball players, S2: Postural recommendations for wheelchair basketball players.

## References

[1] Finley M.A., Rodgers M.M. (2004). Prevalence and identification of shoulder pathology in athletic and nonathletic wheelchair users with shoulder pain: A pilot study. J. Rehabil. Res. Dev..

[2] Samuelsson K.A., Tropp H., Gerdle B. (2004). Shoulder pain and its consequences in paraplegic spinal cord-injured, wheelchair users. Spinal Cord..

[3] Curtis K.A., Roach K.E., Applegate E.B., Amar T., Benbow C.S., Genecco T.D. (1995). Reliability and validity of the Wheelchair User’s Shoulder Pain Index (WUSPI). Spinal Cord.

[4] Curtis K.A., Roach K.E., Applegate E.B., Amar T., Benbow C.S., Genecco T.D. (1995). Development of the Wheelchair User’s Shoulder Pain Index (WUSPI). Spinal Cord.

[5] Curtis K.A., Black K. (1999). Shoulder pain in female wheelchair basketball players. J. Orthop. Sports Phys. Ther..

[6] Ballinger D.A., Rintala D.H., Hart K.A. (2000). The relation of shoulder pain and range-of-motion problems to functional limitations, disability, and perceived health of men with spinal cord injury: A multifaceted longitudinal study. Arch. Phys. Med. Rehabil..

[7] Fullerton H.D., Borckardt J.J., Alfano A.P. (2003). Shoulder pain: A comparison of wheelchair athletes and nonathletic wheelchair users. Med. Sci. Sports Exerc..

[8] Curtis K.A. (1997). Prevention and treatment of wheelchair athletic injuries. Athl. Ther. Today.

[9] Nyland J., Robinson K., Caborn D., Knapp E., Brosky T. (1997). Shoulder rotator torque and wheelchair dependence differences of National Wheelchair Basketball Association players. Arch. Phys. Med. Rehabil..

[10] Fairbairn J.R., Huxel Bliven K.C. (2019). Incidence of shoulder injury in elite wheelchair athletes differ between sports: A critically appraised topic. J. Sport Rehabil..

[11] Tuakli-Wosornu Y.A., Mashkovskiy E., Ottesen T., Gentry M., Jensen D., Webborn N. (2018). Acute, and Chronic Musculoskeletal Injury in Para-Sport: A Critical Review. Phys. Med. and Rehabil..

[12] Crespo-Ruiz B.M., Del Ama-Espinosa A.J., Gil-Agudo A.M. (2011). Relation between kinematic analysis of wheelchair propulsion and wheelchair functional basketball classification. Adapt. Phys. Activ. Q.

[13] Pérez-Tejero J., Martínez-Sinovas R., Rossignoli I. Shoulder pain in Spanish wheelchair basketball players. Proceedings of the 15th European Congress of Physical and Rehabilitation Medicine and 45th National Congress of the Spanish Society of Physical and Rehabilitation Medicine.

[14] De Witte A.M., Berger M.A., Hoozemans M.J., Veeger D.H., van der Woude L.H. (2017). Effects of offense, defense, and ball possession on mobility performance in wheelchair basketball. Adapt. Phys. Activ. Q.

[15] Vanlandewijck Y., Theisen D., Daly D. (2001). Wheelchair propulsion biomechanics: Implications for wheelchair sports. Sports Med..

[16] García-Gómez S., Pérez-Tejero J. (2017). Baloncesto en silla de ruedas: Influencia del dolor del hombro en gestos deportivos. J. Sport Psych..

[17] Curtis K.A., Dillon D.A. (1985). Survey of wheelchair athletic injuries: Common patterns and prevention. Spinal Cord.

[18] Mutsuzaki H., Tachibana K., Shimizu Y., Hotta K., Fukaya T., Karasawa M., Wadano Y. (2014). Factors associated with deep tissue injury in male wheelchair basketball players of a Japanese national team. Sports Med. Arthrosc. Rehabil. Technol..

[19] Shimizu Y., Mutsuzaki H., Tachibana K., Tsunoda K., Hotta K., Fukaya T., Wadano Y. (2017). A survey of deep tissue injury in elite female wheelchair basketball players. J. Back Musculoskelet. Rehabil..

[20] Akınoğlu B., Kocahan T. (2017). Characteristics of upper extremity’s muscle strength in Turkish national wheelchair basketball player’s team. J. Exerc. Rehabil..

[21] Lim K.B., Yoo J., Lee H.J., Lee J.H., Kwon Y.G. (2014). Evaluation of pain and ultrasonography on the shoulder in poliomyelitis wheelchair basketball players. Korean J. Sports Med..

[22] Tsunoda K., Mutsuzaki H., Hotta K., Tachibana K., Shimizu Y., Fukaya T., Wadano Y. (2016). Correlates of shoulder pain in wheelchair basketball players from the Japanese national team: A cross-sectional study. J. Back Musculoskelet. Rehabil..

[23] Cools A.M., Johansson F.R., Borms D., Maenhout A. (2015). Prevention of shoulder injuries in overhead athletes: A science-based approach. Braz. J. Phys. Ther..

[24] Asker M., Brooke H.L., Waldén M., Tranaeus U., Johansson F., Skillgate E., Hollow L.W. (2018). Risk factors for, and prevention of, shoulder injuries in overhead sports: A systematic review with best-evidence synthesis. Br. J. Sports Med..

[25] Silvestri J. (2018). Shoulder preservation in spinal cord injury: One clinic’s approach to treatment and prevention. Curr. Phys. Med. Rehabil. Rep..

[26] Curtis K.A., Tyner T.M., Zachary L., Lentell G., Brink D., Didyk T., Pacillas B. (1999). Effect of a standard exercise protocol on shoulder pain in long-term wheelchair users. Spinal Cord.

[27] Mulroy S.J., Thompson L., Kemp B., Hatchett P.P., Newsam C.J., Lupold D.G. (2011). Strengthening and optimal movements for painful shoulders (STOMPS) in chronic spinal cord injury: A randomized controlled trial. Phys. Ther..

[28] Van Straaten M.G., Cloud B.A., Morrow M.M., Ludewig P.M., Zhao K.D. (2014). Effectiveness of home exercise on pain, function, and strength of manual wheelchair users with spinal cord injury: A high-dose shoulder program with telerehabilitation. Arch. Phys. Med. Rehabil..

[29] Nawoczenski D.A., Ritter-Soronen J.M., Wilson C.M., Howe B.A., Ludewig P.M. (2006). Clinical trial of exercise for shoulder pain in chronic spinal injury. Phys. Ther..

[30] Satyavanshi A., Pattnaik M., Mohanty P. (2017). Comparison of two types of strengthening exercises in upper limbs for improvement of wheelchair propulsion in paraplegics. J. Novel Physiother. Rehabil..

[31] Wilroy J., Hibberd E. (2017). Evaluation of a shoulder injury prevention program in wheelchair basketball. J. Sport Rehabil..

[32] Bergamini E., Morelli F., Marchetti F., Vannozzi G., Polidori L., Paradisi F., Traballesi M., Cappozzo A., Delussu A.S. (2015). Wheelchair propulsion biomechanics in junior basketball players: A method for the evaluation of the efficacy of a specific training program. BioMed. Res. Int..

[33] Soo Hoo J. (2019). Shoulder Pain and the Weight-bearing Shoulder in the Wheelchair Athlete. Sports Med. Arthrosc. Rev..

[34] Warner M.B., Wilson D., Heller M.O., Wood D., Worsley P., Mottram S., Webborn N., Veeger D., Batt M. (2018). Scapular kinematics in professional wheelchair tennis players. Clinic. Biomech..

[35] Armstrong R., Waters E., Moore L., Riggs E., Cuervo L.G., Lumbiganon P., Hawe P. (2008). Improving the reporting of public health intervention research: advancing TREND and CONSORT. J. Public Health.

[36] World Medical Association (2000). Declaration of Helsinki Ethical Principles for Medical Research Involving Human Subjects. Bull. World Health Organ..

[37] García-Gómez S., Pérez-Tejero J., Ocete C., Barakat R. (2017). Expert’s opinion of a home-based exercise program for shoulder pain prevention: Application in wheelchair basketball players. Psychol Soc. Educ..

[38] García-Gómez S., Pérez-Tejero J., García B., Barakat R. (2016). Validity and Reliability of the Shoulder Pain Index for Wheelchair Basketball Players. J. Sport Psychol..

[39] Hawkins R.J., Kennedy J.C. (2018). Impingement syndrome in athletes. Am. J. Sports Med..

[40] Neer C.S. (1983). Impingement lesions. Clin. Orthop. Relat. Res..

[41] Hughes P. (2011). The Neer sign and Hawkins-Kennedy test for shoulder impingement. J. Physiother..

[42] Hughes P.C., Taylor N.F., Green R.A. (2008). Most clinical tests cannot accurately diagnose rotator cuff pathology: A systematic review. Aust. J. Physiother..

[43] Jobe F.W., Moynes D.R. (1982). Delineation of diagnostic criteria and a rehabilitation program for rotator cuff injuries. Am. J. Sports Med..

[44] Rossi D.M., Resende R.A., da Fonseca S.T., de Oliveira A.S. (2018). Scapulothoracic kinematic pattern in the shoulder pain and scapular dyskinesis: A principal component analysis approach. J. Biomech..

[45] Gillooly J.J., Chidambaram R., Mok D. (2010). The lateral Jobe test: A more reliable method of diagnosing rotator cuff tears. Int. J. Shoulder Surg..

[46] Riddle D.L., Rothstein J.M., Lamb R.L. (1987). Goniometric reliability in clinical setting: shoulder measurements. Phys. Ther..

[47] Mulroy S.J., Gronley J.K., Newsam C.J., Perry J. (1996). Electromyographic activity chair propulsion of shoulder muscles during wheelchair propulsion by paraplegic persons. Arch. Phys. Med. Rehabil..

[48] Rosnow R.L., Rosenthal R. (1996). Beginning Behavioral Research: A Conceptual Primer.

[49] Kerby D.S. (2014). The simple difference formula: An approach to teaching nonparametric correlation. Innov. Teach..

[50] Cohen J. (1992). A power primer. Psychol. Bull..

[51] Østerås H., Torstensen T.A., Østerås B. (2010). High dosage medical exercise therapy in patients with long-term subacromial shoulder pain: A randomized controlled trial. Physiother. Res. Int..

[52] Van Straaten M.G., Cloud B.A., Zhao K.D., Fortune E., Morrow M.M. (2017). Maintaining Shoulder Health after Spinal Cord Injury: A Guide to Understanding Treatments for Shoulder Pain. Arch. Phys. Med. Rehabil..

[53] Cratsenberg K.A., Deitrick C.E., Harrington T.K., Kopecky N.R., Matthews B.D., Ott L.M., Coeytaux R.R. (2015). Effectiveness of exercise programs for management of shoulder pain in manual wheelchair users with spinal cord injury. J. Neurol. Phys. Ther..

[54] Jacobs P.L., Nash M.S. (2004). Exercise recommendations for individuals with spinal cord injury. Sports Med..

[55] Mulroy S., Haubert L.L., Eberly V., Conners S., Weiss W. (2017). Effectiveness of two intervention programs to prevent shoulder pain after spinal cord injury. Arch. Phys. Med. Rehabil..

[56] Frontera W.R., Slovik D.M., Dawson D.M. (2006). Exercise in Rehabilitation Medicine.

[57] Figoni S.F. (2009). Spinal Cord Disabilities: Paraplegia and Tetraplegia ACSM’s Exercise Management for Persons with Chronic Diseases and Disabilities.

[58] Gauthier C., Brosseau R., Hicks A.L., Gagnon D.H. (2018). Feasibility, Safety, and Preliminary Effectiveness of a Home-Based Self-Managed High-Intensity Interval Training Program Offered Long-Term Manual Wheelchair Users. Rehabil. Res. Pract..

[59] Yüksel M., Sevindi T. (2018). Examination of Performance Levels of Wheelchair Basketball Players Playing in Different Leagues. Sports.

[60] Dutton R.A. (2019). Medical and Musculoskeletal Concerns for the Wheelchair Athlete: A Review of Preventative Strategies. Curr. Sports Med. Rep..

