# Supplementary material for: Effect of a Home-based Exercise Program on Shoulder Pain and Range of Motion in Elite Wheelchair Basketball Players: A Non-Randomized Controlled Trial
Source: Sports (Basel). 2019 Jul 24;7(8):180. doi: 10.3390/sports7080180 (PMC6723715; doi:10.3390/sports7080180)
Supplement: Supplementary file 1 [file sports-07-00180-s001.pdf]

SHOULDER EXERCISE PROGRAM TO PREVENT SHOULDER INJURIES IN WHEELCHAIR BASKETBALL PLAYERS

Exercises will be performed in seating position, 3 times per week. If you feel any significant pain or discomfort, you must contact the physiotherapist in charge.

The time of execute the exercise program is from 25 to 30 minutes

A) ACTIVE MOVEMENTS

Description: With relax arms, move shoulders in different directions: (1) Raise arms out in front, (2) Circular movements, (3) Raise out both arms to the side above the head at the same time and (4) with the arms separated of the body move the arms forward and backward. Considerations: Be careful with trunk movements. 8 repetitions of each one.

(1) 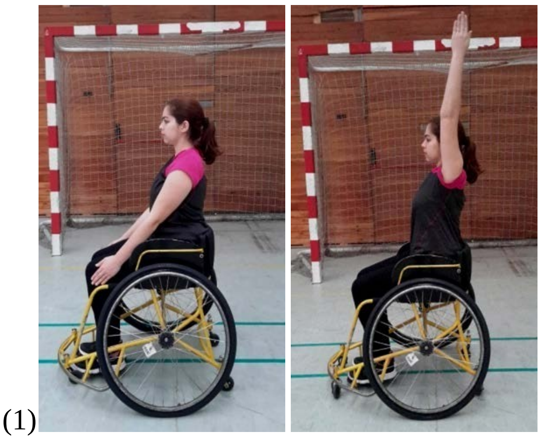

(2) 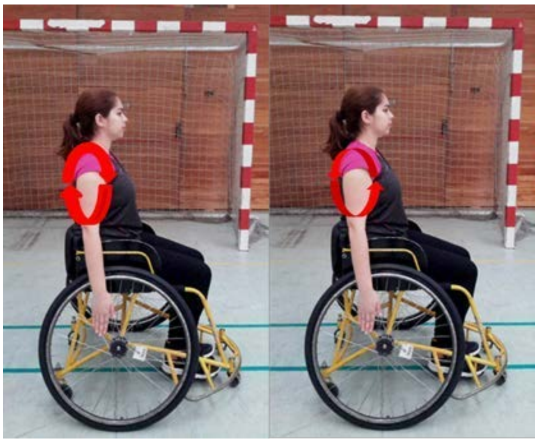

(3) 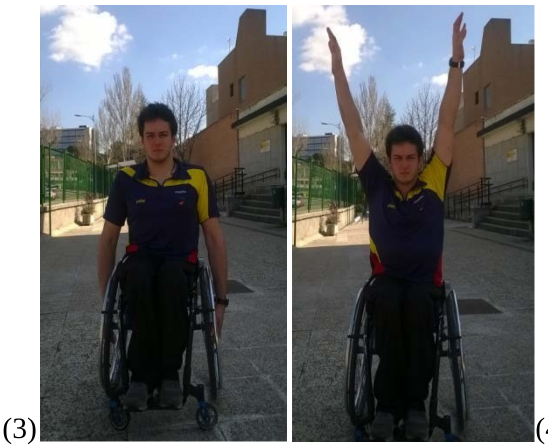

(4) 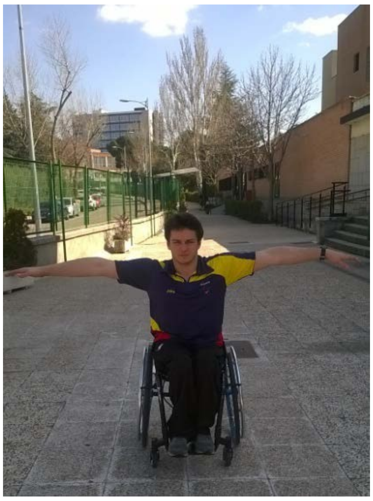

B) STRENGTHENING: 3 sets 10 repetitions with 45s of rest between sets.

1. - Serratus anterior

Description: Seated in upright position with resistive band attached to object in backward, hold the band with elbow in 90 degrees. Extended elbow and flexion the shoulder at the same time. Return to start position slowly. Repeated with opposite side. Considerations: Try to not move your trunk to do the exercise.

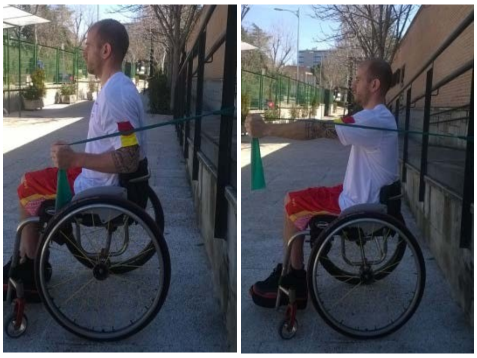

Figure 1. Strengthening of serratus anterior.

2. - Scapula retraction and depression

Description: Seated in upright position with resistive band attached in front of the wheelchair, hold the band with elbow extended. Move arms down and backward then return slowly to starting position. Repeated with opposite side.
Considerations: Try to not move your trunk to do the exercise.

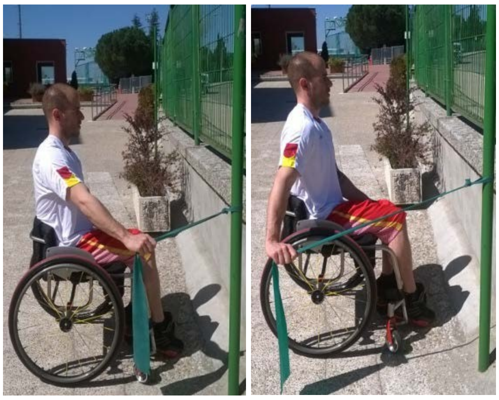

Figure 2. Strengthening of scapula retraction and depression.

3.- External rotation

Description: Wheelchair at 90º of a wall with resistance band secure on the opposite side, hold the band with arm in neutral position and the elbow in 90º. Pull the band out of the side away of the bodied. Hold the position and return slowly to initial position. Repeated with opposite side.
Considerations: Avoid separate arm of the body to make the movement.

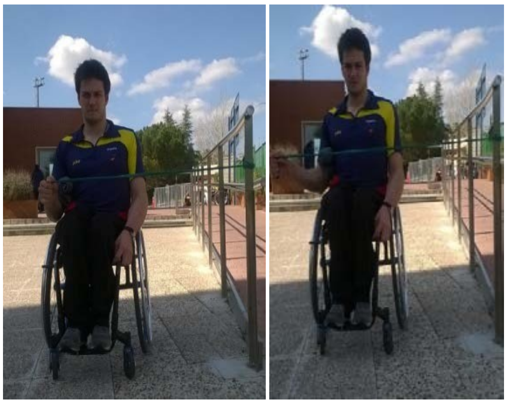

Figure 3. Strengthening of external rotation

4. - Internal rotation

Description: Wheelchair at 90º of a wall with resistance band secure on the same side, hold the band with arm in neutral position and the elbow in 90º. Pull the band inward to your stomach. Hold the position and return slowly to initial position. Repeated with opposite side.
Considerations: Avoid separate arm of the body to make the movement.

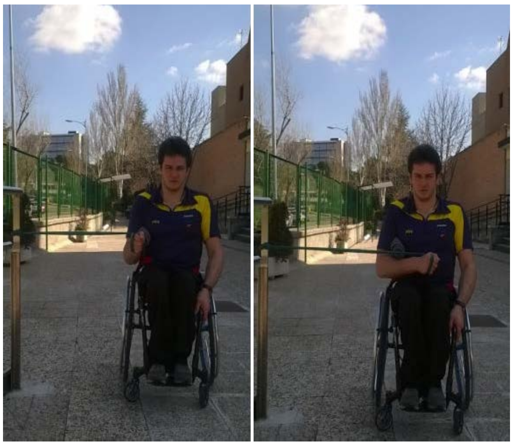

Figure 4. Strengthening of internal rotation

5. - Shoulder Adduction

Description: Seated in upright position with resistance band attached above head height, hold the band with arm aligns at a height of shoulder with elbow straight. Pull the band with the hand trying to touch the opposite knee with the elbow straight and thumb pointing down. Repeated with opposite side. Considerations: Avoid separate arm of the body to make the movement. Considerations: Avoid doing neck flexion and don't move the upper trunk.

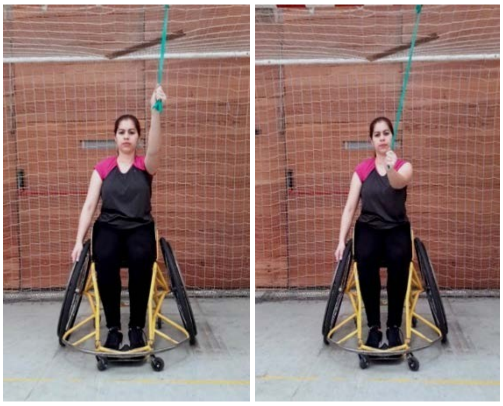

Figure 5. Strengthening of shoulder adduction

C) STRETCHING (5 repetitions of each one, hold each stretch for 20/30s. Rest 15s)

1. - Trapezius superior

Description: In upright position, use one hand to stabilize the trunk holding the wheelchair. With the opposite hand, lean away the necks to the opposite side. Considerations: Avoid neck rotation.

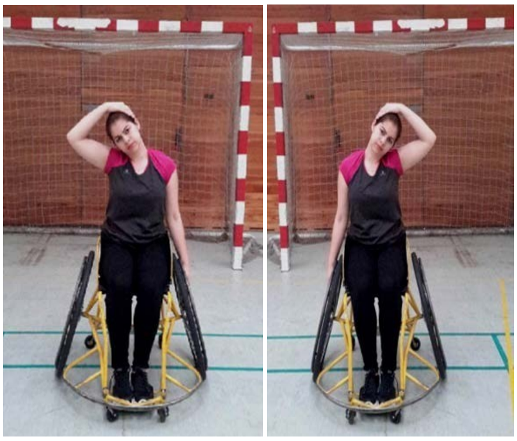

Figure 1. Stretching of trapezius superior

1. Posterior of the shoulder

Description: Seated in upright position, pull arm slowly around the front of the upper body using the opposite hand above the elbow to stretch. Considerations: Avoid neck rotation.

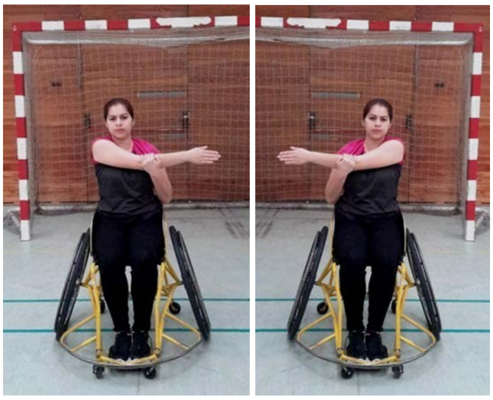

Figure 2. Stretching of posterior muscles of the shoulder

3. - Pectoral muscle

Description: Position wheelchair in a frame, shoulder 90º of abduction and elbow less than 90º holding in the border. Rotate upper body slowly away from the frame until the stretch and hold. Considerations: Hold the position and do not support with opposite arm.

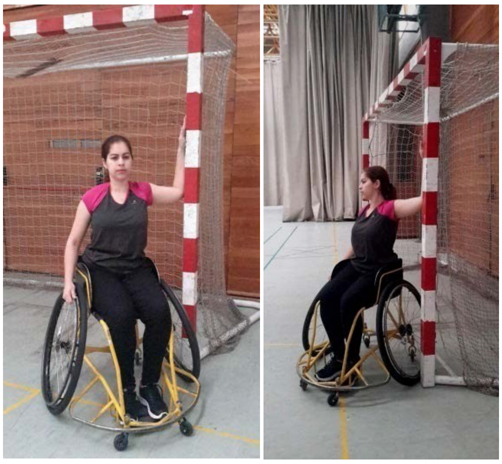

Figure 3. Stretching of pectoral muscle

4. - Biceps braquial (long head)

Description: Position wheelchair in a frame, shoulder in extension and external rotation 90º of abduction and elbow less than 90º holding in the border of the frame. Rotate upper trunk slowly. Consideration: Be careful, it's necessary the depression and retraction of the scapula during the stretching.

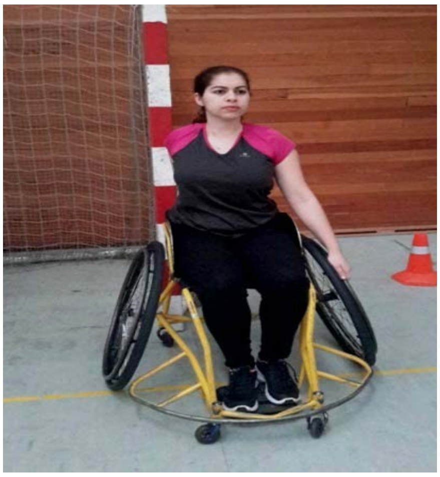

Figure 4. Stretching of braquial muscle

5. - Trapezius (Medial and inferior portion)

Description: Seated in upright position, stretch upper limb holding hands over the shoulders. Considerations: Hold the position the time required, avoid neck rotation.

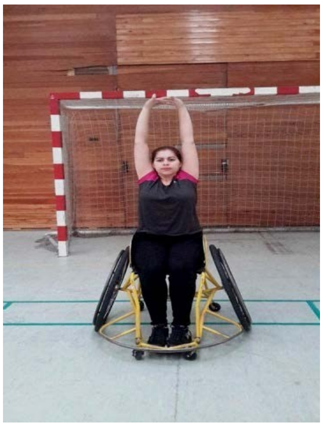

Figure 5. Stretching of medial and inferior portion of the trapezius

If you feel **unusual pain or discomfort**, contact with the physiotherapist or the research staff.

Francisco Javier Rosa Gaspar
fjrosa_87@hotmail.com/ feddf.bsrsalud@gmail.com Saleky García Gómez saleky@gmail.com

POSTURAL RECOMMENDATIONS FOR WHEELCHAIRS BASKETBALL PLAYERS

SPANISH NATIONAL SELECTION 2016

Shoulder injuries could be as a consequence of overuse and postural alterations in the daily living activities o sport activities. In this regard, it is necessary to have an adecuate posture to reduce stress to perform movements regardless of the activity being carried out. In order to reduce the load on the shoulder, as well as the possible damage to the spine to prevent injury and muscle imbalances we will use guidelines of postural hygiene. This uses the minimum tension and rigidity, thus allowing maximum efficiency and minimum energy expenditure.

When lying flat

The ideal lying position is side with both knees bent and place the pillow between your legs to keep the spine stable, because of that to be placed upside down or mouth above you can create tension at the lumbar level and alter the lumbar curvature; however if being side put a pillow at the level of the pelvis and stand slightly sideways diminishes the risk to develop disorders of the spine.

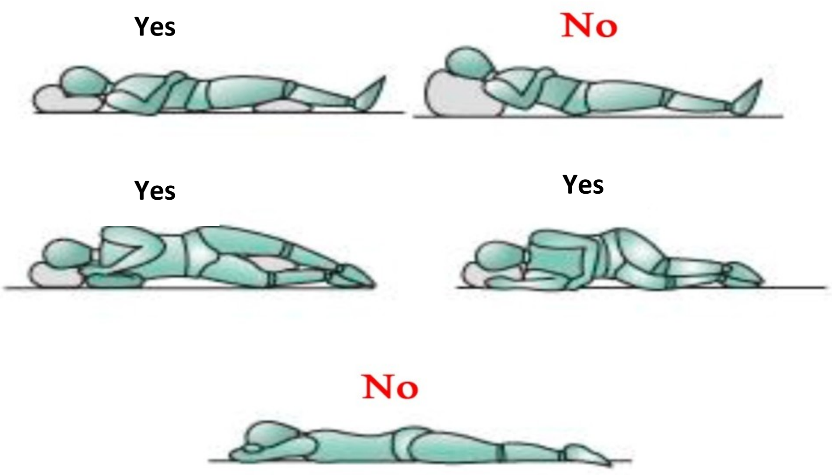

In the case of being upside down can use a thin pillow and ensure that the cervical and dorsal column keeps that same angle to the stand. Waking up is good to stretch to adapt our muscle tone to the activity. To lift us we must Flex the knees and turn the trunk to incorporate us side. Mattress and pillow features influence the positions adopted when these lying down. The mattress must be firm and straight, in such a way that it fits the curvature of his spine. Regarding the use of the pillow, take into account that we will use it to accommodate our body and prevent muscle strain.

Sittting position

Both for the activities of everyday life how sporting keep sitting posture / a must maintain an upright posture, although it must be dynamic and allow the functionality of joints, the backing of the same secure the back respecting normal curvatures.

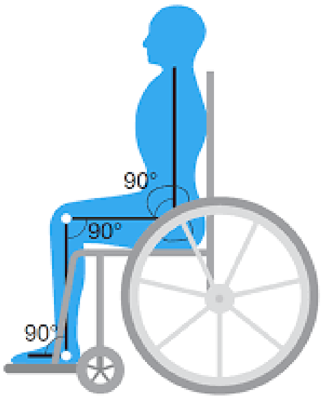

Good posture provides stability, comfort and distributes the pressure to areas of lower risk of producing scars, in addition to influencing the effective propulsion of the wheelchair. You should avoid partial turns, the correct way to do is to turn the whole body at the same time and perform movements when you hold this position for a long time.

Working with a computer

The screen should place it at a safe distance and at its height, placing the keyboard to be low so as not to lift the shoulders and allow the forearms to rest on the table. The wrist

and forearms should be straight and aligned with the keyboard; you could use a palm rest.

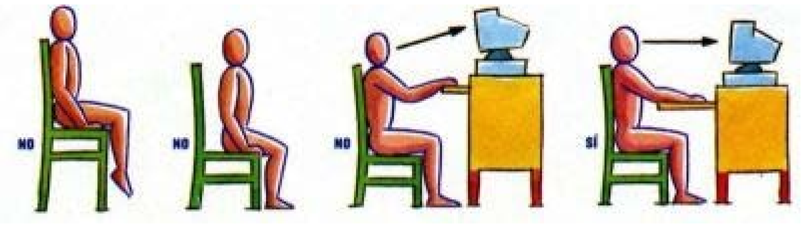

Driving

You must position the seat in such a way that you can reach the pedals without having to stretch your legs, and rest on the backrest. The knees should be at or above hip level.

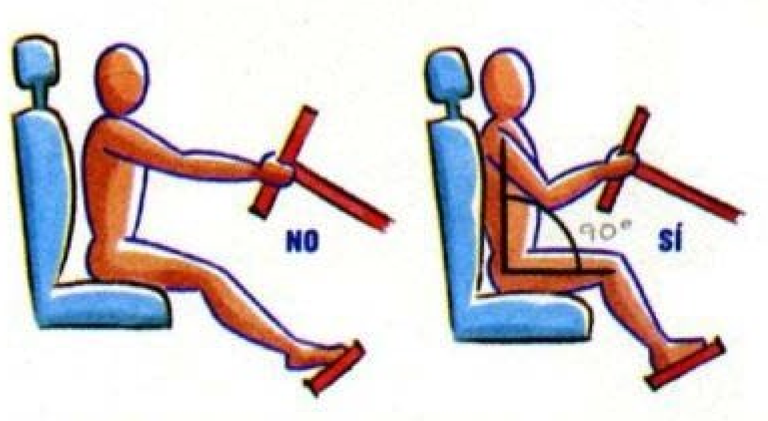

Lifting objects

The objects should be placed at the height of the chest, with the elbows flexed to ensure this as close to the body as possible. Avoid doing overuse lifting objects inappropriately.

These are general recommendations, since depending on the type of injury and level of injury in the case of spinal cord injuries could condition some postures.

If you have any question contact with the physiotherapist or the research

staff. Francisco Javier Rosa Gaspar fjrosa_87@hotmail.com

Saleky García Gómez saleky@gmail.com
